# SING: Subgraph search In Non-homogeneous Graphs

**DOI:** 10.1186/1471-2105-11-96

**Published:** 2010-02-19

**Authors:** Raffaele Di Natale, Alfredo Ferro, Rosalba Giugno, Misael Mongiovì, Alfredo Pulvirenti, Dennis Shasha

**Affiliations:** 1 Dipartimento di Matematica ed Informatica, Università di Catania, Catania, Italy; 2 Courant Institute of Mathematical Sciences, New York University, New York, USA

## Abstract

**Background:**

Finding the subgraphs of a graph database that are isomorphic to a given query graph has practical applications in several fields, from cheminformatics to image understanding. Since subgraph isomorphism is a computationally hard problem, indexing techniques have been intensively exploited to speed up the process. Such systems filter out those graphs which cannot contain the query, and apply a subgraph isomorphism algorithm to each residual candidate graph. The applicability of such systems is limited to databases of small graphs, because their filtering power degrades on large graphs.

**Results:**

In this paper, SING (Subgraph search In Non-homogeneous Graphs), a novel indexing system able to cope with large graphs, is presented. The method uses the notion of *feature*, which can be a small subgraph, subtree or path. Each graph in the database is annotated with the set of all its features. The key point is to make use of feature locality information. This idea is used to both improve the filtering performance and speed up the subgraph isomorphism task.

**Conclusions:**

Extensive tests on chemical compounds, biological networks and synthetic graphs show that the proposed system outperforms the most popular systems in query time over databases of medium and large graphs. Other specific tests show that the proposed system is effective for single large graphs.

## Background

Graphs naturally model a multitude of complex objects in the real world. A chemical compound can be represented by a graph where atoms are vertices and bonds are edges. Biological networks model the complex of interactions among components in cells, (e.g. proteins, genes, metabolites). Social networks, the web, the water system and the power grid are all represented by graphs. A basic operation is the search of a query graph in a target graph or, more generally, in a database of graphs. Searching a molecular structure in a database of molecular compounds is useful to detect molecules that preserve chemical properties associated with a well known molecular structure. This can be used in screening and drug design. Searching subnetworks in biological networks helps to identify conserved complexes, pathways and motifs among species, and assist in the functional annotation of proteins and other cell components. The problem of searching for a query graph in a target graph is called *subgraph isomorphism *and is known to be NP-complete. Since the subgraph isomorphism test is expensive, screening all graphs of a large database can be unfeasible. Recently, indexing techniques for databases of graphs have been developed with the purpose of reducing the number of subgraph isomorphism tests involved in the query process. In a *preprocessing *phase the database of graphs is analyzed and an index is built. A query is processed in two phases. In the *filtering *step the index is used to discard the graphs of the database which cannot contain the query, producing a small set of *candidate *graphs. The set of candidates is then verified (*verification *step) by a subgraph isomorphism algorithm and all the resulting matches are reported.

Most graph indexing tools are based on the concept of *feature*. Depending on the particular system, a feature can be either a small graph [1-3], a tree [4] or a path [5,6]. The filtering property is based on checking whether the features of the query are contained in each target graph. In the preprocessing phase the database of graphs is scanned, the features are extracted from each graph and stored in the index data structure. During the filtering phase, the features are extracted from the query and the index is probed in order to discard all graphs which do not contain some feature of the query.

Existing indexing techniques are effective on databases of small graphs but they become unfeasible when applied to huge graphs [1,2]. The reason is that features that may be rare in small graphs are likely to be found in enormous graphs just by chance. This implies that filtering systems based only on the presence or number of features are not effective for large graphs. Moreover the subgraph isomorphism test over a large graph is extremely expensive. Unfortunately, alternative indexing systems which do not make use of features [4,7] show similar problems on large graphs.

To make the verification phase faster, GraphGrep [5] stores all the feature occurrences of each graph, and discards the part of the graph which does not contain features of the query thus restricting the search to small portions of the target graph. However, this produces a large index which is more difficult to manage and can lead to a reduction in filtering performance. Furthermore, the features of the query often occur in many parts of the graphs, reducing the filtering power.

In this paper, a novel approach to cope with large graphs is proposed. The present approach makes use of paths as features. In contrast to systems that use more complex features such as subgraphs or subtrees, our index includes all paths of bounded length. The position of a feature within the graph is considered. This additional information is used to both improve the filtering power and guide the verification phase allowing an effective pruning of the search tree. In contrast to GraphGrep, only the starting point of a feature is stored and bit arrays are used to reduce the index size. Furthermore this information is used to optimize the verification phase. Notice that this approach cannot be used for graph features since graphs have no starting points. Although a similar approach could be used for tree features (using the roots as starting points), the resulting preprocessing time would be higher since enumerating subtrees is much more expensive than enumerating paths. Despite using path features, our system is effective in capturing the topology of graphs and it is shown to perform better than existing systems in terms of query processing time, while keeping the size of the index comparable. An extensive experimental analysis on real and synthetic data shows that the proposed system is efficient and effective on both databases of small graphs and single large graphs.

### Preliminaries

This paper considers undirected node-labeled graphs. However, the concepts introduced in what follows can be easily extended to edge-labeled and directed graphs. An undirected labeled graph (in what follows simply a graph) is a 4-tuple *g *= (*V*, *E*, Σ, *l*) where *V *is the set of vertices, *E *⊆ *V *× *V *is the set of edges (a symmetric binary relation on *V*), Σ is the alphabet of labels and *l*: *V *→ Σ is a function which maps each vertex onto a label. If *e *= (*v*_1_, *v*_2_) is an edge, then *v*_1 _and *v*_2 _are called its *endpoints*. We set *size*(*g*) = |*E*| and indicate with  the set of all possible graphs. A graph *g*_1 _= (*V*_1_, *E*_1_, Σ, *l*_1_) is said to be a subgraph of another graph *g*_2 _= (*V*_2_, *E*_2_, Σ, *l*_2_) iff *V*_1 _⊆ *V*_2 _and *E*_1 _⊆ *E*_2_.

Given two graphs *g*_1 _= (*V*_1_, *E*_1_, Σ, *l*_1_), *g*_2 _= (*V*_2_, *E*_2_, Σ, *l*_2_) an *isomorphism *between *g*_1 _and *g*_2 _is a bijection *ϕ*: *V*_1 _→ *V*_2 _so that:

• (*u*, *v*) ∈ *E*_1 _⇔ (*f*(*u*), *f *(*v*)) ∈ *E*_2_

• *l*_1_(*u*) = *l*_2_(*f *(*u*))∀* u *∈ *V*_1_

A *subgraph isomorphism *between *g*_1 _and *g*_2 _is an isomorphism between *g*_1 _and a subgraph of *g*_2_. A graph *g*_1 _is said to be isomorphic to another graph *g*_2 _if there exist an isomorphism between *g*_1 _and *g*_2_. For the sake of simplicity we say also that *g*_1 _is equivalent to *g*_2 _and write *g*_1 _≈ *g*_2_. Notice that ≈ is an equivalence relation on . A graph *g*_1 _is said to be subgraph isomorphic to another graph *g*_2 _if there exist a subgraph isomorphism between *g*_1 _and *g*_2_. In this case we say that *g*_1 _is *contained *in *g*_2 _and write *g*_1 _≾ *g*_2_.

In this paper, the following two problems will be discussed:

*First_query_occurrence *problem: Given a database of *n *graphs *D *= {*g*_1_, *g*_2_, ..., *g*_*n*_} and a query graph *q*, executing the query *q *on *D *is equivalent to finding all graphs *g *of *D *such that *q *is subgraph isomorphic to *g*. In the following we assume, without loss in generality, that all graphs of *D *and the query graph, share the same alphabet Σ.

*All_query_occurrences *problem: Given a database of *n *graphs *D *= {*g*_1_, *g*_2_, ..., *g*_*n*_} and a query graph *q*, executing the query *q *on *D *is equivalent to finding all subgraph isomorphisms between *q *and elements of *D*. We will make extensive use of the notion of feature. Features are formally introduced by the following definition.

**Definition 1 ***Let **be the set of all possible graphs in a given alphabet of labels. A set *ℱ *is a *set of features on *iff there exists a binary relation is_a_feature *⊆ ℱ × *such that the following property holds (*graph upward monotonicity):

∀ *f *∈ ℱ, *q*, *g *∈ ;

*is_a_feature*(*f*, *q*) ∧ *q *≾ *g *→ *is_a_feature*(*f*, *g*)

In what follows, *is_a_feature*(*f*, *g*) is expressed by saying that *g contains f*.

Every set of features defines a *pruning rule *for the subgraph isomorphism problem:

**Pruning rule 1 ***If is_a_feature*(*f*, *q*) *and ¬ is_a_feature*(*f*, *g*) *then q cannot be subgraph isomorphic to g*.

Examples of set of features are:

• The set *Paths*_≤ *k *_of all labeled paths of length ≤ *k*. Here a labeled path is the sequence of labels.

• The set *Subtrees*_≤ *k *_of all labeled subtrees of depth ≤ *k*.

• The set *Subgraphs*_≤ *k *_of all labeled subgraphs of size ≤ *k*.

This paper considers the set of features *Paths_occ*_≤ *k *_of pairs (*p*, *n*), where *p *is a labeled path of length ≤ *k *and *n *is a lower bound on the number of occurrences of *p *in the given graph. The corresponding pruning property asserts that if the query graph *q *contains at least *n *occurrences of a given labeled path *p *and *g *does not contain at least *n *occurrences of *p*, then *q *cannot be subgraph isomorphic to *g *and *g *can be pruned.

Notice that in all above examples if a feature *f *is a subfeature of a given feature *f' *of *g *then *f' *is also a feature of *g*. The following definition formalizes this notion.

A *downward monotonic *set of features is a partially ordered set of features (ℱ; ) such that,

∀ *f*, *f' *∈ ℱ, *g *∈ ,

*f ** f' *∧ *is_a_feature*(*f'*, *g*) → *is_a_feature*(*f*, *g*)

**Figure 1:**
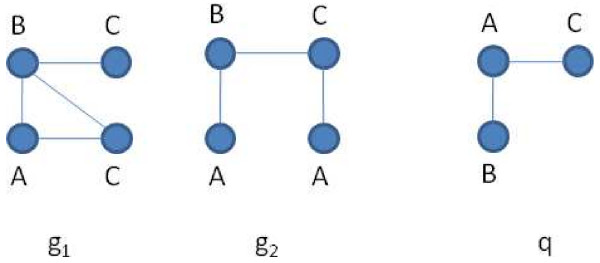
**Graph samples**. A database of two graphs *g*_1_, *g*_2 _and a query *q*. *q *≾ *g*_1 _but *q *⋨ *g*_2_.

For instance *Paths*_≤ *k *_is a downward monotonic set of features with respect to the subsequence relation between labeled paths. *Paths_occ*_≤ *k *_is downward monotonic with respect to the number of occurrences. However it is not downward monotonic with respect to the subsequence relation. Indeed in Figure 1, (ABC,2) is a feature of *g*_1 _but (AB,2) is not a feature of *g*_1_.

A downward monotonic set of features allows an additional optimization in the pruning process: the pruning rule can be restricted only to maximal features *f *in the query. This means that no other feature *f' *in the query can be strictly greater than *f *in the partial order of features.

### Related work

Graph indexing systems are based on a filter-and-verification scheme which includes two main phases: (1) preprocessing: an index is built by scanning the database; (2) query processing: the index is probed to efficiently answer the query. The query processing is divided in two sub-steps: filtering and matching. The filtering step prunes all graphs of the database which cannot contain the query graph, generating a set of candidate graphs. The matching step executes a subgraph isomorphism algorithm on all candidate graphs. All graph indexing systems except CTree [4] and GCoding [7] use the concept of feature. Table 1 synthesizes the characteristics of the main graph indexing tools. In the next subsections we briefly survey feature-based and non-feature-based systems respectively.

**Table 1:** Graph indexing systems

System	Features	Data mining	All matches
GraphGrep	Path	No	Yes
gIndex	Graphs	Yes	No
FGIndex [2]	Graphs	Yes	No
GDIndex [3]	Graphs	No	Yes
TreePi [8]	Tree	Yes	No
Tree+*δ *[9]	Tree+graphs	Yes	No
CTree	-	No	No
GCoding	-	No	Yes
SING	Path	No	Yes

Review of the main graph indexing systems.

#### Feature-based graph indexing systems

All feature-based graph indexing systems are characterized by choosing a set of features ℱ and apply a Pruning rule 1 to features of ℱ. To prune as many graphs as possible, the graph indexing systems consider a set of features *F*_*q *_⊆ ℱ such that each feature *f ∈ F*_*q *_is contained in *q*, and prune all graphs *g ∈ D *which do not contain some feature in *F*_*q*_. The filter-and-verification scheme is performed in the following way:

• **Preprocessing: **each graph of the database is examined off-line in order to extract all features of ℱ which are contained in the graph. An inverted index is generated, which maps each feature *f *∈ ℱ into the set *graph_set*(*f*) of all graphs containing *f*.

• **Query processing:**

**-Filtering: **The given query *q *is examined in order to extract a suitable set *F*_*q *_⊆ ℱ of features contained in *q*. A set of candidate graphs *C *is then computed by .

**-Matching: **Each candidate graph is examined in order to verify that the given query is subgraph isomorphic to it. If the All_query_occurrences problem must be solved, then an exhaustive enumeration of all distinct subgraph matches is executed.

The differences among the various graph indexing systems lie mainly in the choice of the sets ℱ and *F*_*q*_. ℱ can be a set of bounded-size graphs, trees or paths. Since the number of features can be very high, some graph indexing systems select a restricted feature set from the database. For example gIndex [1] selects frequent subgraphs of bounded size. This operation requires the performance of an expensive graph data mining step during the preprocessing phase. A possible choice for *F*_*q *_is *F*_*all *_= {*f *∈ ℱ|*is_a_feature*(*f*, *q*)}. If ℱ is an ordered feature set, *F*_*q *_can be chosen, without loss in pruning power, to be the set *F*_*max *_of all maximal features in *F*_*all*_. It is also possible to choose any set *F*_*q *_: *F*_*max *_⊆ *F*_*q *_⊆ *F*_*all*_. This is the choice made in SING.

Some indexing systems consider also more effective pruning rules based on the number of feature occurrences [5,6] and the distances between features [8]. Some systems define compact representations of the index [2,6]. A description of the various indexing systems follows, with a discussion of the positive and negative aspects of the various choices.

**Graph features**. Some systems such as gIndex [1], GDIndex [3] and FGIndex [2] use graphs as features. They consider a set of features ℱ = , where  is the universe of graphs and  is the partition of  induced by graph isomorphism. All isomorphic graphs are considered as a single feature represented by their equivalence class. The main advantage of using graph features is that they are more suitable to capture the topological structure of graphs. Consequently they tend to produce fewer candidates.

Unfortunately, the number of graph features grows exponentially with the graph size, leading to a large index which degrades the performance of the preprocessing and filtering phases. To solve this problem, gIndex [1] and GDIndex [3] choose as features a set of frequent subgraphs. gIndex [1] considers also the concept of *discriminative subgraphs *to further reduce the number of features. All these approaches require the performance of an expensive data mining step in the preprocessing phase, leading to a loss of efficiency. Moreover, when it comes to coping with large graphs the mining step may become impractical. FGIndex uses a small index resident in main memory, and stores the remaining index in secondary storage. The authors of FGIndex use a novel concept of *δ-tolerance closed frequent subgraph *to distinguish from main-memory-resident features and secondary-memory-resident ones. When the query cannot be performed using only the main-memory-resident index, the main-memory index is used to identify the blocks of the secondary memory index to be loaded. To avoid expensive disk accesses, a small set of maximal features which cover the whole query graph is selected.

GDIndex enumerates all induced subgraphs contained in each graph of the database. It organizes all the features in a DAG representing the partial order relation  among features. The size of the index is reduced by avoiding redundancy. Each feature is associated with the set of graphs containing it and not containing any ancestor-feature in the DAG. During the filtering phase, the set of graphs containing a feature can be deduced by the feature-DAG. Enumerating all subgraphs of a graph is very expensive, therefore this approach can be used only on databases of very small graphs.

**Tree features**. Tree features are easier to manage since the tree-isomorphism problem can be solved in polynomial time. TreePi [8] is the first attempt to use trees as features. The authors describe a linear-time algorithm for computing the canonical labeling of a tree. They experimentally show that tree features capture the topological structure well enough. Therefore, using them may result in a good compromise between efficiency and effectiveness of filtering. As shown by authors, a unique center can be defined for a tree. Consequently the distance (shortest path) between pairs of features in a graph can be computed. TreePi uses an additional pruning rule based on distances between features to improve the quality of the match. More precisely, this pruning rule is based on the observation that for a query graph to be subgraph isomorphic to a target graph, the distance between each pair of query vertices cannot be less than the distance between corresponding vertices in the target graph. Tree+*δ *[9] uses as features both trees and a restricted class of small graphs to improve the filtering performance. As for graphs, enumerating all trees of bounded size still produces a large number of features. Consequently, a restricted set of features needs to be selected by an expensive data mining step.

**Path features**. GraphGrep [5] and GraphFind [6] consider as features all paths of length up to *l*_*p *_(usually 4). Formally a k-length *path *of a graph *G *= (*V*, *E*, Σ, *l*) is an ordered sequence of vertices (*v*_1_, *v*_2_, ..., *v*_*k*_) ∈ *V*^*k *^such that (*v*_*i*_, *v*_*i*+1_) ∈ *E *for 1 ≤ *i *≤ *k *- 1. We say that a path is *simple *if all of its vertices are distinct. A *path feature *on Σ is an ordered sequence of labels (*a*_1_, *a*_2_, ..., *a*_*k*_) where *a*_1_, *a*_2_, ..., *a*_*k *_∈ Σ.

Given a graph *g *= (*V*, *E*, Σ, *l*) and a path feature *f *= (*a*_1_, *a*_2_, ..., *a*_*k*_) ∈ Σ^*k*^, *f *is said to be *contained in g*, in symbols *is_a_feature*(*f*, *g*), if there is a simple path (*v*_1_, *v*_2_, ..., *v*_*k*_) ∈ *V*^*k *^such that *l*(*v*_*i*_) = *a*_*i *_for 1 ≤ *i *≤ *k*. In this case (*v*_1_, *v*_2_, ..., *v*_*k*_) is called a *path occurrence *of *f *starting from *v*_1_.

Fixed an integer *l*_*p *_> 0, the set  is a partially ordered feature set with respect to the relation  defined by:

(*a*_1_, ... *a*_*n*_)  (*b*_1_, ..., *b*_*m*_) if *n *≤ *m *and *a*_*i *_= *b*_*i *_∀*i *= 1 ... *n*.

Therefore, a Pruning rule 1 can be used to select candidate graphs.

To improve the quality of filtering, the number of path occurrences of each path feature is stored in an inverted index. When a query *q *is processed, a set *F*_*q *_of path features is extracted from it and the number of occurrences of each path feature in the query is compared to the corresponding number of occurrences of the same path feature in each graph of the database. A graph is a candidate if the number of occurrences of each path feature in it is greater than the corresponding number in the query.

GraphGrep also stores the location of each path occurrence in the graphs of the database. Moreover, for each candidate graph, it prunes all parts of the graph which do not contain any path feature of the query. This choice produces an improvement of the matching phase. However the resulting index size is quite large.

The choice of using path features in GraphGrep leads to a very efficient preprocessing phase. On the other hand, it limits the filtering power since paths cannot fully synthesize the topology of graphs.

#### Non-feature based graph indexing systems

Recently, two non-feature-based graph indexing systems have been proposed. They have been shown to outperform many feature-based indexing systems, probably because they are able to better capture the structure of graphs.

CTree [4] organizes the graphs of the database in a R-tree-like data structure. The leaves represent single graphs while internal nodes represents sets of graphs synthesized in graph structures called *closure graphs*. The closure graph of a set of graphs is obtained in the following way. All graphs in the set are aligned by a fast approximate algorithm, called *Neighbor Biased Mapping*. The vertices of the closure graph are labeled by the sets of labels of the corresponding aligned vertices. Similarly, the edges of the closure graphs are the union of aligned edges. When a query is given, an approximate matching algorithm with no-false-negatives is executed on the closure graphs of the tree in a top-down fashion. When the closure graph of a node has a negative response, all the subtrees rooted at that node are pruned and all its leaf graphs are discarded. The remaining graphs are the candidates, and they can be verified by an exact matching algorithm.

Despite the flexibility and filtering power of CTree [4], its filtering efficiency is limited since the execution of the approximate matching algorithm is expensive and needs to be applied to many closure graphs.

GCoding [7] uses the eigenvalue properties of the adjacency matrix for pruning graphs. In particular, it makes use of the *Interlacing theorem *which bounds the eigenvalues of the adjacency matrices of matching graphs. In the preprocessing phase, all the graphs of the database are scanned. For each vertex *v *of a given graph, a *vertex signature *is computed. This computation involves its label, its neighbor's labels together with the higher eigenvalues of the adjacency matrix of the tree rooted on *v *and representing all n-length paths starting from *v*. The vertex signatures of a graph are then merged to form the *graph signature*. Finally the graph signatures are organized in a B-tree-like structure for efficient search. When a query *q *is given, the vertex and the graph signatures of *q *are computed. The graph signature is used to identify in the B-tree a first set of candidate graphs. Than, a second set of candidate graphs is selected from the first one by discarding all graphs whose vertex signatures do not match the vertex signatures of the query. The correspondence between graph signatures and vertex signatures is defined by applying the Interlacing theorem.

Thanks to its coding strategy based on eigenvalues, GCoding [7] allows a compact representation of the index. However the computation of eigenvalues is expensive, leading to a slower preprocessing phase. Finally, the loss of information introduced by the chosen coding produces a less effective pruning compared to CTree [4].

## Results and Discussion

### Approach

The proposed approach is based on a new feature-locality-based pruning rule that reduces the set of candidates resulting from the application of Pruning rule 1. The new pruning rule captures the structure of the graphs much better, leading to a strong reduction of candidates. Locality information is also used to reduce the search space of the verification phase. Our concept has been inspired by Treepi [8], which uses the concept of distance between features, requiring the computation of all-pair-distances. In certain cases, especially when it comes to deal with large graphs, the approach of Treepi is computationally expensive. Enumerating all trees produces an explosion of the number of features that must be reduced by a data mining step. This leads to increase the preprocessing time as well as keep the filtering performances limited, due to the small number of feature selected. Moreover, Treepi requires the computation of the pairwise distances between features. To limit the preprocessing and filtering time, a small number of features need to be selected. Consequently, an high number of candidates is produced. In contrast, SING considers all paths starting from a node. It requires much less computation producing low preprocessing and filtering time. Moreover, SING is able to capture the topology of the tree induced by a node, using simple paths. Consequently it requires a lower number of features and avoid the expensive feature selection process. Consider the graphs in Figure 1. It is easily verifiable that *q *is subgraph isomorphic to *g*_1 _but not to *g*_2_. *q *contains the features (*A*, *B*) and (*A*, *C*) and they are also contained in both *g*_1 _and *g*_2_. Based on these features the graph *g*_2 _cannot be pruned. Note that the occurrences of both features in *q *start from the same vertex. The same situation holds in *g*_1 _but not in *g*_2_. More precisely in *g*_2 _there is no vertex from which occurrences of both features start. Consequently vertex labeled A of *q *cannot match with any vertex of *g*_2_, which can be pruned. The following statements formalize this concept. They are immediate consequences of the definition of subgraph isomorphism. Let *start*(*f*, *g*) be the set of vertices *v *such that an occurrence of *f *starts from *v *in *g*.

**Statement 1 ***Given two graphs q *= (*V*_*q*_, *E*_*q*_, Σ, *l*_*q*_) *and g *= (*V*_*g*_, *E*_*g*_, Σ, *l*_*g*_), let *ϕ*: *V*_*q *_→ *V*_*g *_*be a subgraph isomorphism between q and g. For each vertex v∈ V*_*q *_*the following holds:*

{*f ∈ *ℱ|*v *∈ *start*(*f*, *q*)} ⊆ {*f ∈ *ℱ |*ϕ *(*v*) ∈ *start*(*f*, *g*)}.

**Statement 2 ***Given two graphs q, g. If q *≾ *g then for each vertex v of q must exist at least a vertex u in g so that*

*{f ∈ ℱ|v ∈ start*(*f*, *q*)} ⊆ {*f *∈ ℱ|*u *∈ *start*(*f*, *g*)}.

Statement 2 suggest a more effective way to prune the graph database. Given a candidate graph *g*, for each vertex *v *of the query graph *q*, there exists a vertex *u *of *g *such that each feature starting from *v *also starts from *u*. Consequently, if for some vertex of *q *there is no such corresponding vertex *u*, *g *can be pruned. Statement 1 gives a method to reduce the search space of the matching algorithm. That is, it introduces a more restrictive condition on the matching pairs of vertices. A detailed description of each phase of the proposed graph indexing system is given in Section Methods.

### Experimental Analysis

This section compares the proposed system to the most popular tools. Three different dataset classes are used. Tests on real data coming from a database of small molecules (DTP AIDS Antiviral Screen) and a database of biological networks labeled with gene expressions are performed. We evaluate SING on large graphs by generating a synthetic scale-free network of 2000 nodes and executing several queries of sizes ranging from 4 to 16.

The proposed system was implemented in C++ and compiled with the GNU compiler gcc 3.3. Experimental analysis was performed on an Intel Xeon with 2 GB of memory using Linux OS. The executable used to perform all the experiments here reported is available as Additional File 1. The other tools used for the comparison are: CTree [4], GCoding [7], gIndex [1] and Tree+Delta [9].

#### Molecular data

Experiments on molecular data were performed over the DTP AIDS Antiviral Screen dataset published by the National Cancer Institute [10]. The complete dataset contains about 42000 chemical compounds. The experiment took three subsets containing respectively 8000, 24000 and 40000 graphs. Each compound corresponds naturally to a graph whose nodes are the atoms labeled with their atomic symbol. Each simple or multiple chemical bond between two atoms is represented by a single edge.

For each database, a set of queries is generated in the following way. Randomly choose a graph *g *of the database and one of its vertices *v*. Starting from *v*, proceed randomly in a breadth-first fashion until a fixed total number *t *of edges is reached. This yields groups of 100 queries, each having a number of edges equal to 4, 8, 16 and 32 respectively.

**Figure 2:**
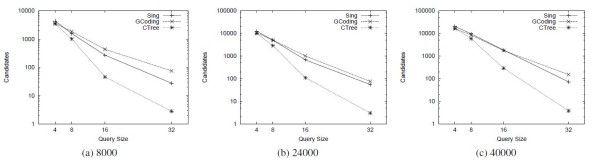
**Candidates**. Number of candidates over databases of molecular compounds.

**Figure 3:**
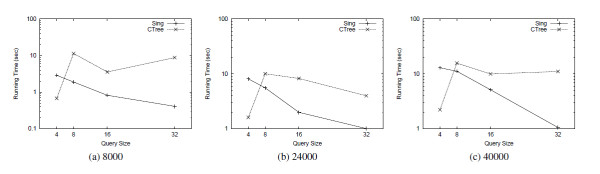
**Query time First_query_occurrence**. Total query time over databases of molecular compounds. The tools solve the First_query_occurrence problem.

**Figure 4:**
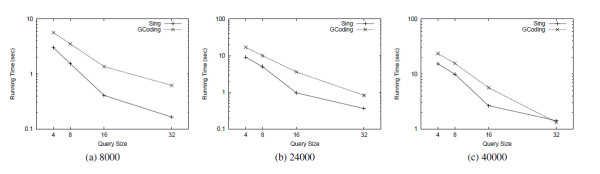
**Query time All_query_occurrences**. Total query time over databases of molecular compounds. The tools solve the All_query_occurrences problem.

Tables 2 and 3 show the comparison results of SING against CTree [4] and GCoding [7] with respect to preprocessing time and index size, respectively. SING builds the index more rapidly than GCoding. Since CTree [4] does not extract the features from the graphs, its preprocessing time is much lower than the other tools. Figure 2 reports the average number of candidates passing the filtering phase on each query group of a given size. Figures 3 and 4 compares SING in terms of average query processing time against CTree [4] and GCoding [7], respectively. As above, tests are performed on query groups of a given size. CTree [4] and GCoding consider First_query_occurrence and All_query_occurrences, respectively.

**Table 2:** Preprocessing time

Database size(kb)	CTree	GCoding	SING
8000	8	642	149
24000	25	1948	452
40000	42	2960	755

Preprocessing time on AIDS molecular compounds. The times are expressed in seconds.

**Table 3:** Index size

Database size	CTree	GCoding	SING
8000	13844	6687	8445
24000	41372	20088	25279
40000	70208	30651	42830

Index Size on AIDS molecular compounds. The sizes are expressed in KB.

Consequently, Figure 3 reports comparisons on the First_query_occurrence problem whereas Figure 4 refers to the All_query_occurrences problem. SING outperforms all the other tools in all tests except 4-size queries. At size 4, CTree [4] outperforms the other tools, but its filtering and matching steps scale less well as size increases. More in detail, CTree [4] uses an expensive filtering process based on an approximated algorithm for subgraph isomorphism. As a matching algorithm it uses a variant of the Ullmann algorithm [11] integrated in the framework, which has been shown to be outperformed by VF2 [12]. The advantage of CTree [4] in the preprocessing phase suggests its employment in applications having a small number query executions per graph. In high query environments, using SING may be more appropriate. Table 4 shows the best-performing tool depending on the number of queries and the query size. The table takes the total of preprocessing and query time into account.

**Table 4:** Winner table

# of queries	size 4	size 8	size 16	size 32
≤ 70	CTree	CTree	CTree	CTree
71-147	CTree	CTree	CTree	**SING**
148-157	CTree	CTree	**SING**	**SING**
≥ 158	CTree	**SING**	**SING**	**SING**

The best-performing system depending on the number of queries of a given size. Experiments on the AIDS database 40,000 molecular compounds. Comparison between SING, CTree and GCoding.

**Figure 5:**
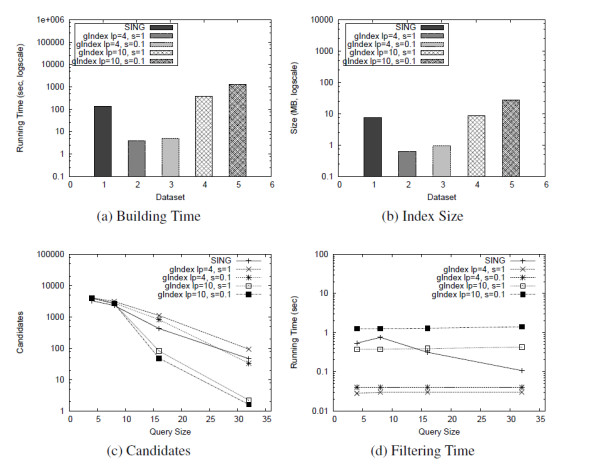
**Comparison with gIndex**. Comparison with gIndex over a dataset of 8000 small molecular compounds.

Since the gIndex [1] data mining step is expensive, especially over large graphs, this system was not able to run on these databases. For example, in the experiments reported in [1] all H atoms, together with their bonds, were deleted. In order to compare SING against gIndex, we generated a dataset of small molecular compounds as follows. From the whole AIDS compounds database, 43 graphs having more than 250 nodes or edges were first discarded. Then, 8000 graphs were selected at random from the resulting dataset. gIndex [1] was executed for 4 different configurations. The the maximum feature size was set to 4 and 10. gIndex [1] uses a support threshold which grows with the feature size. The maximum support threshold was set to 0.1 and 1. Figure 5 reports the results. Here *lp *denotes the maximum feature size and *s *denotes the maximum support threshold. Since gIndex [1] does not perform the matching task, the total query time is not reported.

With *lp *= 4, the building time, the index size and the filtering time are much lower than SING because of the small number of features considered by gIndex [1]. For the same reason, the filtering performances are worse than that with *lp *= 10 (higher number of candidates) and, with the exception of query size 32 with *s *= 0.1, the number of candidates is always higher than SING.

With *lp *= 10 and *s *= 0.1, the index size is comparable (7.5 MB of SING against 8.5 MB of gIndex), but gIndex [1] require 3 times more to build it (391 seconds against 135 seconds of SING). The filtering time of gIndex [1] tends to be constant with respect to the query size. Compared to gIndex [1], SING takes more filtering time on low-size queries. On the other hand SING takes less time when the query size increases. The filtering power of SING and gIndex [1] are comparable over small queries. gIndex [1] produces a smaller number of candidates on larger queries at the expense of a longer preprocessing and filtering time. With *lp *= 10 and *s *= 0.1, gIndex [1] shows a little improvement in pruning power, but the preprocessing performances drop and the filtering time is higher, mainly due to the high number of features considered. We also performed a comparison with Tree+Delta [9], a recently proposed system which uses as features trees and a small set of selected graphs. Tree+Delta [9] performs better than TreePi, consequently, we do not compare SING with TreePi.

**Figure 6:**
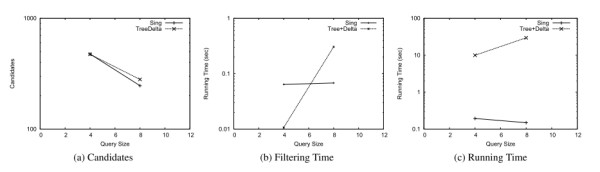
**Comparison with Tree+Delta**. Comparison with Tree+Delta over a dataset of 800 small molecular compounds.

Similarly to what we observed for gIndex [1], Tree+Delta [9] could not run on the whole AIDS dataset. Therefore, in order to perform the comparison with SING, we randomly generated a small dataset of 800 molecular compounds from AIDS database. The size of the graphs in this dataset ranges from 3 nodes and 4 edges to 276 nodes and 284 edges. The evaluation was performed by using 100 queries of size (number of edges) 4 and 100 queries of size 8. The parameters of Tree+Delta [9] was set according to [rif- ariticolo]. More precisely we set the maximum feature size maxL to 10. The size of the index generated by Tree+Delta [9] is 114 kb (74 kb for SING) and the pre-processing time is 11 seconds (the same for SING). As reported in Figure 6a and Figure 6b the number of candidates of Tree+Delta [9] is lower than that produced by SING, (except for query size 4, for which the number of candidates are equal). Furthermore, SING always outperforms Tree+Delta [9] on query processing time. The filtering time of Tree+Delta [9] is about one fold lower than SING for query size 4 and about one fold higher than SING for query size 8. In contrast the filtering time of SING is almost constant, which confirms the good scalability of SING compared with Tree+Delta [9]. Concerning queries of size greater than 8, comparison are not reported since Tree+Delta [9] requires too much time on the average.

#### Transcription networks

To evaluate the performance of SING on large networks, we generated a database of gene networks labeled with discretized gene expressions, based on a transcription regulation network of Escherichia Coli annotated with gene expressions. We extracted The largest connected component from the complete network, available with the supplementary material of [13]. Gene expression profiles of 22 samples of the experiment GDS2825 (freely available from NCBI [14]), concerning the analysis of E. Coli K12 strains adapted to grow in benzalkonium chloride (a commonly used disinfectant and preservative) were used. We discretized each gene expression value by mapping it into a set of 5 levels: very low, low, medium, high, very high. Those levels became the node labels of the regulatory networks.

Following Alon [13], we attempted to identify groups of nodes connected with a given topology and annotated with a certain gene expression level profile. One can use this approach to understand a gene regulation mechanism, by verifying if a given pattern is present in a set of samples, where it occurs and which genes are involved.

**Figure 7:**
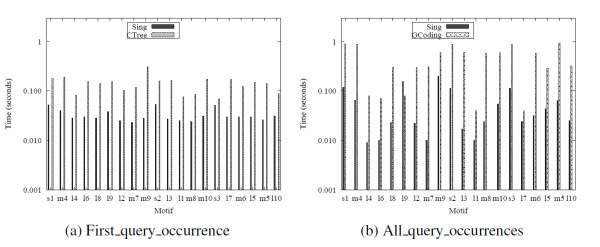
**Results on biological networks**. Query time over biological networks.

We queried the networks database with a set of motifs labeled with gene expression levels using motifs found by U. Alon et. al. [13]. Each vertex was labeled with the gene expression level "very high". Preprocessing time and index size are reported in Table 5. Figure 7 reports the total processing time of each query, named as in [13]. SING outperforms the other methods in query processing time.

**Table 5:** Preprocessing over biological networks

Tool	Index size (KB)	Preprocessing time (sec)
SING	1252	23
CTree	292	1.3
GCoding	85	101

Preprocessing time and index size over biological networks

#### Synthetic data

**Figure 8:**
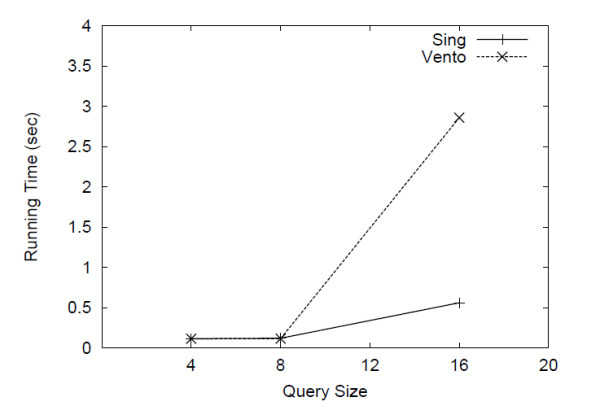
**Results on a single graph**. Query time over a single large graph.

To evaluate the performance of SING over a single large graph, we generated a scale free network of 2000 nodes having about 4000 edges. The network was generated adding the edges one by one in the following way. Each new edge is connected to an existing node of the network with probability proportional to the degree of that node. This procedure guarantee that the produced network has a power law degree distribution. The labels were assigned distributing 8 different labels at random (with uniform distribution) over the network nodes. We used as queries three different sets of 10 queries with size (number of edges) respectively 4, 8 and 16. The queries were generated at random using the same procedure discussed above (see par. "Molecular data"). We then evaluated the query time of SING against VF2 [12]. We did not consider the other tools because the filtering phase is useless for a single graph which contains the query, and the verification phase is usually performed by VF2 (the algorithms that do not use VF2 clearly perform worse). SING generated a 1.2 MB index in 35 seconds. The query time is reported in Figure 8. For small queries (size 4 and 8), SING and VF2 show similar performances whereas for query size 16, SING outperforms VF2.

#### Protein-protein interaction networks

Protein-protein interaction networks (PPI) are in general very complex to manage by most of graph minining tools [1,2]. Indeed, PPI networks are scale free and the degree of their nodes follows the power law. These networks are characterized by the presence of nodes, called hubs, carrying hundreds of connections (edges). In these graphs most of the nodes are connected through a few hubs. The matching phase for this kind of networks is very heavy for most of the available tools.

**Figure 9:**
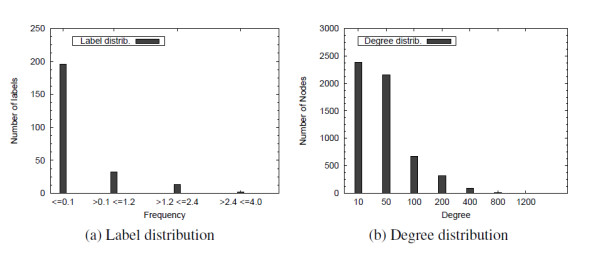
**Label and degree distributions**. Label distribution in the human network (clusters) and degree distribution on the yeast network.

To illustrate the performance of SING over these networks we consider the whole human PPI network containing 7824 nodes and 28303 edges. As a set of queries we used 282 protein complexes of yeast. Protein nodes were retrieved from SGD, whereas edges were inferred from PPI data by BioGRID [15]. We executed all-pair-BLAST on the set of proteins of yeast and human, and then clustered them by single-linkage clustering. To avoid grouping dissimilar proteins in the same cluster, we applied a score cutoff of 40 bits. Moreover we set 100 as the maximum cluster size. The human network and the yeast complexes were then labeled by the ID of the clusters, obtaining 1251 labels. The distribution of labels in this network, is described in Figure 9a. Notice that, approximately 200 labels show a frequency of about 0.1% (i.e. the 20% of labels appears only about 5 times in the network). Removing the complexes with a number of edges less than 2 produces a set of 209 yeast complexes, whose size ranges from 2 to 1290 edges (the transcription factor complex).

Feature based tools such as gIndex [1], Tree+Delta [9], do not perform on large graphs since they were not designed for such purpose. Therefore, we compared SING only with VF2 [12].

The human network was preprocessed by SING in 613 seconds producing an index of 9556 Kb. We then queried the human network with the yeast complexes. We found that only 7 yeast complexes have at least one match in human. Table 6 reports the total query processing time performed by SING and VF2 when execute in this small set of 7 queries and in the set of remaining queries (the complexes that don't have matches in human). As expected, SING outperforms VF2 when applied on queries which are not contained in the target network. When applied on queries with at least one match, SING and VF2 perform nearly equal query time.

**Table 6:** Query time (in sec.) of matching complexes in the human-yeast experiment

QueryName	VF2	SING
CAF	2,928	2,982
Elg1 RFC like	2,984	2,996
Piccolo NuA4 histone acetyltransferase	2,959	2,980
SNARE 31201	2,952	2,983
methionyl glutamyl tRNA synthetase	2,960	3,032
nascent polypeptide associated	2,952	2,976
tubulin	3,034	2,994

Preprocessing time and index size over biological networks

**Figure 10:**
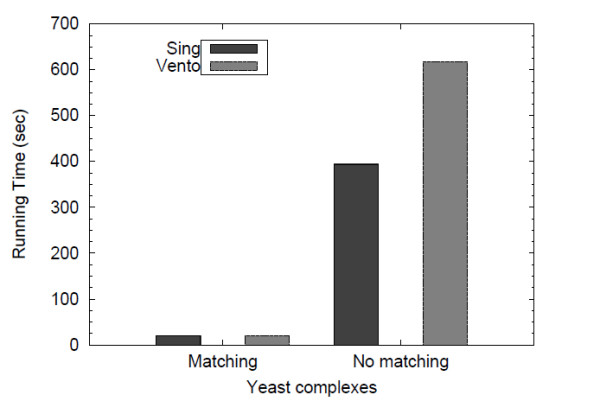
**Query time yeast vs. human**. Results of querying the human network with a set of yeast complexes. The query time has been distinguished for matching and non-matching complexes.

Figure 10 reports the total query processing time required by SING and VF2 on both a set of matching queries and a set of non-matching ones. SING outperforms VF2 when applied on queries which are not contained in the target network. When applied on the remaining queries, SING and VF2 show a similar behavior. This is because the high number of labels (1251) compared to the number of nodes (7824) makes the localization easy for both VF2 and SING.

To compare the algorithms on a more difficult environment, we considered the whole yeast network (downloaded from BioGRID [15]). This network contains 5589 nodes and 92835 edges. Figure 9b describes the degree distribution for this network. 8 different labels were assigned to the nodes at random with uniform distribution.

100 queries of size 4 and 8 and 50 queries of size 16 were randomly extracted from the network. Since a query can have a huge number of matches, we report the running time after at most 100 matches were reached.

**Figure 11:**
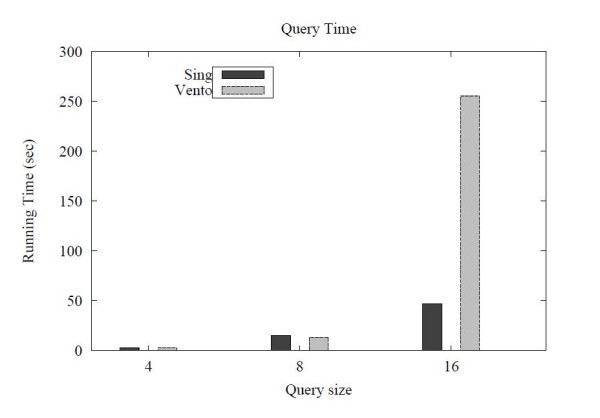
**Results on the yeast network**. Query time over the yeast network (random, 8 labels).

**Figure 12:**
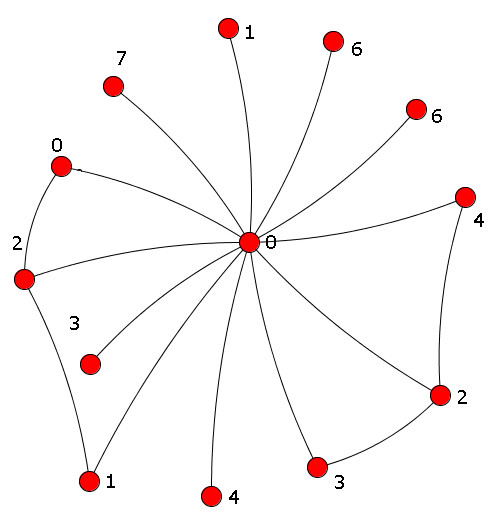
**A sample of non-matching query for VF2**. A sample of non-matching query for VF2.

Figure 11 depicts the SING and VF2 performances relating to query of size 4, 8 and 16. SING and VF2 are comparable for the first two sizes of query, while for query of size 16 SING outperforms VF2. Analyzing the results more in detail, we found a significant case in which the behavior of SING and VF2 is totaly different. For a query of size 16, VF2 spends about 2.3 hours to execute it, while SING is able to process it in 8 seconds. This query is reported in Figure 12. Even if this query is composed by only 13 nodes and 16 edges, there is a central hub with 12 edges. When VF2 try to match the the central hub of the query with a hub in the target network (let us call it *H*), the combinatorial exploration of its neighborhood can be very expensive. In contrast, in many cases SING can verify early that the two hubs cannot be matched. For instance, if for the path 0-4-2-3, contained in the query and starting from the central node, there is not a corresponding path in the graph starting from *H*, SING discard the match and continue, avoiding the expensive verification of the neighborhood.

## Conclusions

**Figure 13:**
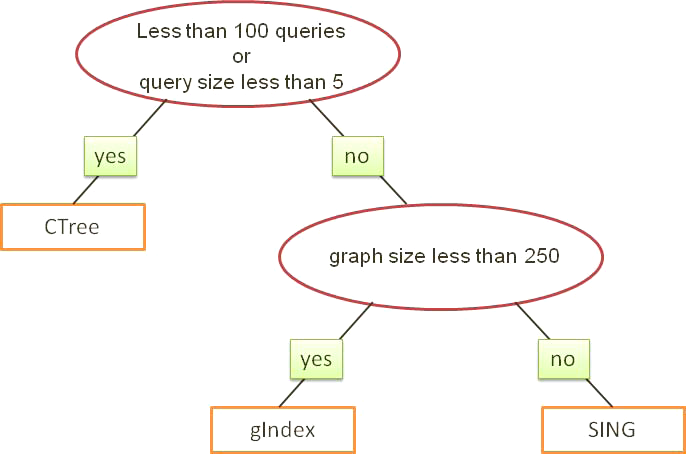
**Decision tree**. A decision tree showing the best performing system on different scenarios. SING is the best choice on applications where a lot of queries need to be processed on databases of medium and large graphs.

We have introduced a new algorithm for graph search called SING and compared it with its most popular competitors. SING performs filtering to discard graphs and additional semantic filtering during the detailed graph search. Our experiments suggested that CTree [4] should be used when few (under 100) queries need to be performed on the graphs. On databases of small graphs (under 250 nodes), gIndex [1] may give better filtering at the expense of longer construction cost compared with SING. SING is best when a high number of queries will be performed on databases of large graphs. Figure 13 provides a decision tree which suggests the system to be used over different scenarios.

## Methods

In this section we report a detailed description of the proposed system. Our indexing system is based on the classical Pruning rule 1 and the new pruning rule introduced in Section Results. The three steps of the filter-and-verification scheme are discussed separately.

### Preprocessing

**Figure 14:**
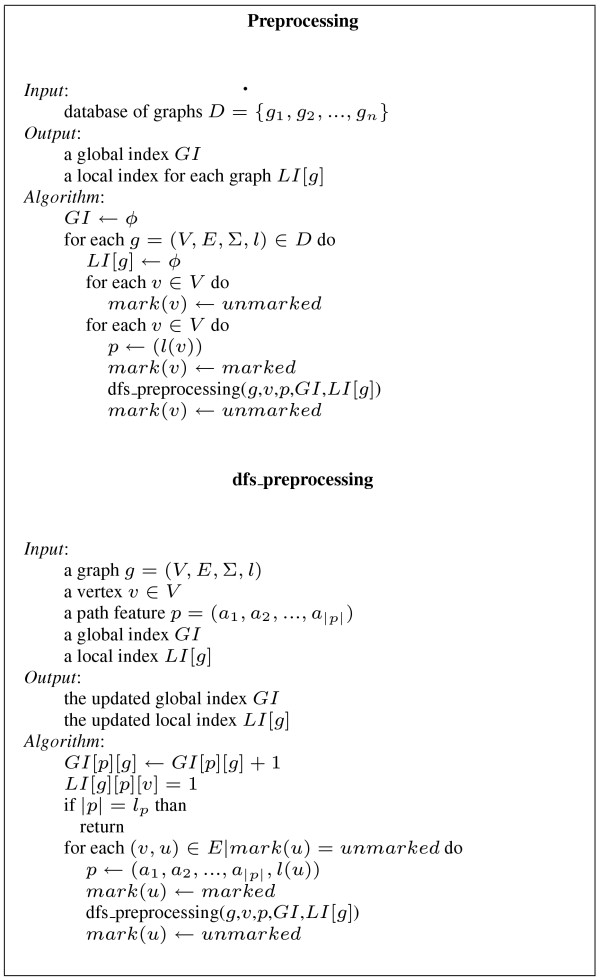
**Preprocessing**. Preprocessing algorithm.

The preprocessing phase constructs two data structures (for details, see Figure 14). First, it constructs a global index that maps each path feature to the set of graphs containing it (*GI *[*f*]) and the number of occurrences of that path feature in each graph. The second index maps each feature to the set of starting vertices of all its occurrences. *LI *[*g*] [*f*] [*v*] = 1 if an occurrence of the feature *f *in the graph *g *starts from *v*. Otherwise *LI *[*g*] [*f*] [*v*] = 0.

Construction visits all graphs of the database and enumerates, using a depth-first strategy, all feature paths contained in each graph. That is, starting from each vertex it visits all paths of depth at most *l*_*p*_, where *l*_*p *_is a fixed threshold (usually *l*_*p *_≤ 10, by default *l*_*p *_= 4).

The structures *GI *and *LI *can be implemented using hash tables. Therefore the average complexity of the above algorithm in the database *D *is , where *d*_*m *_is the maximum degree of the vertices and *v*_*a *_is the average number of vertices in graphs of *D*. In our implementation the structures *GI *and *LI *are binary trees, so the complexity is  in the worst case.

In the implementation, *LI *[*g*] [*p*] is represented as a bit array whose size is equal to the number of nodes of *g*. This choice both reduces the index space and produces faster filtering (Section Second step filtering below gives details).

### Filtering

**Figure 15:**
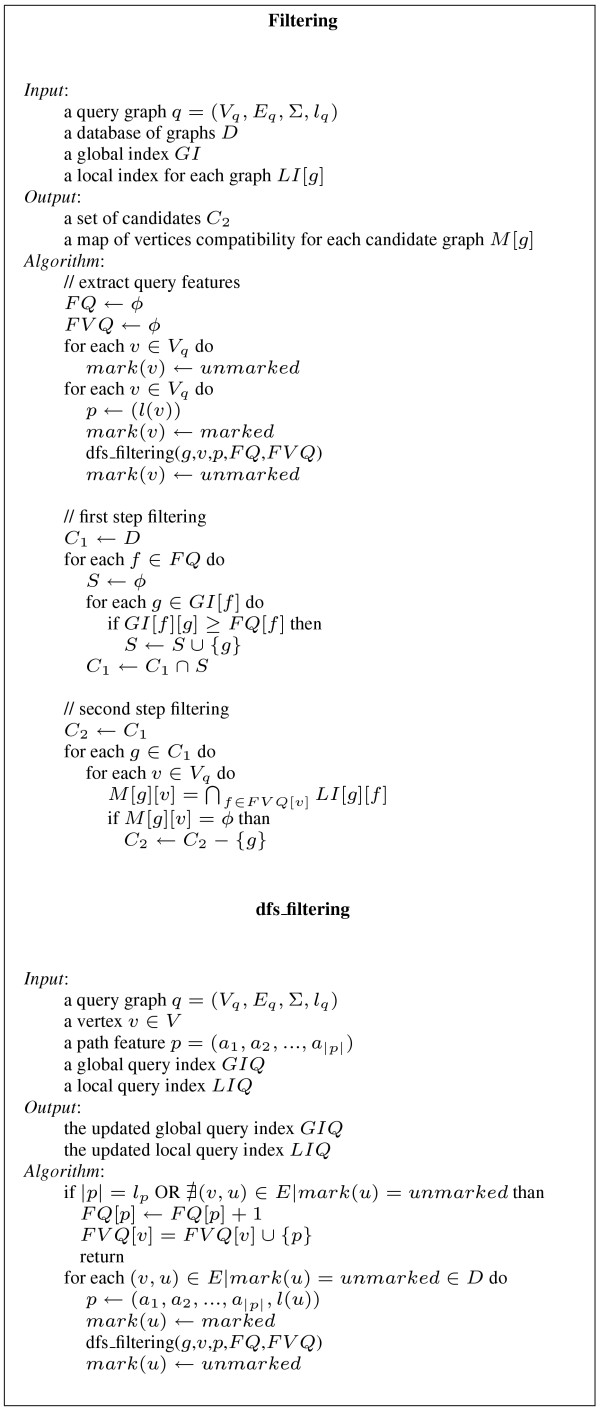
**Filtering**. Filtering algorithm.

The filtering phase applies Pruning rule 1 and Statement 2 to prune the graphs of the database which cannot contain the query (see Figure 15 for details). Structure *FQ *is the set of features contained in the query, extracted by using a depth-first search strategy similar to the one used in preprocessing. The procedure looks only for maximal paths within the query, discarding all paths that are prefixes of a larger path. FVQ associates with each vertex the set of features starting from that vertex. This is used in the second filtering step.

The first step of the pruning procedure computes the set *C*_1 _= ⋂_*f*∈*FQ *_{*g ∈ GI *[*f*]: *GI *[*f*] [*g*] ≥ *FQ *[*f*]}.

This retains graphs only if they have all features in the query. In addition, if a graph does not have at least as many occurrences of each feature as the query does, it is discarded.

If *FQ *and *FV Q *are represented using hash tables, the average complexity of extracting query features is , where *d*_*m *_is the maximum degree of the vertices and *V*_*q *_is the set of query vertices. The complexity of the first step filtering is  using hash tables. In practice the complexity is lower since the set of graphs associated to a feature is lower than |*D*| and not all possible path features occur in the database.

#### Second step filtering

The first step of filtering takes into account only the occurrences of a feature in the database graphs. The second step filtering uses locality information to further prune the database. For each graph *g *which passes the first step filtering test, a mapping between query vertices and vertices of *g *is computed by the following procedure. Let *v *be a vertex of the query and *FV Q *[*v*] be the previously computed set of features starting from *v*. The algorithm computes *M *[*g*] [*v*] = ⋂_*f*∈*FVQ *[*v*] _*start*(*f*, *g*) as the set of vertices of the graph *g *compatible with *v*. If for some vertices *v *we have *M *[*g*] [*v*] = ∅ then the graph *g *is discarded. Statement 2 guarantees the correctness of this second filtering step.

Since *LI *is implemented using bit arrays, the set *M *[*g*] [*v*] can be efficiently computed by the logical AND operation. The complexity is  if *M *is implemented by either vectors or hash tables.

In practice, this is an extremely fast operation on modern hardware.

### Matching

To each candidate graph, the VF2 [12] subgraph matching algorithm is applied. VF2 is a combinatorial search algorithm which spawns a search tree by branching states and makes use of a set of feasibility rules to prune the search. Every state of VF2 consists of a partial match between the query and the target graph. Starting from an initial state consisting of an empty match, VF2 produces a sequence of states by increasing the size of the partial match. Each step generates a new state by adding a pair of corresponding vertices to the partial match. When the partial match cannot be extended, the algorithm backtracks. To decide if two vertices can be matched, VF2 uses a set of topological feasibility rules and a semantic feasibility rule (largely based on label comparison).

SING replaces the semantic compatibility criterion between nodes with a more efficient test to reduce the breadth of the search tree. As described in Section Second step filtering, for each vertex *v *of the query, a set *M *[*g*] [*v*] of compatible vertices of the graph *g *is computed. *M *[*g*] [*v*] represents the set of vertices of *g *which can be matched to *v*. In fact, by definition if a vertex *v' *of the graph *g *does not belong to *M *[*g*] [*v*], there is at least one feature *f *such that *v ∈ start*(*f*, *q*) and *v' *∉ = *start*(*f*, *g*). It follows by Statement 1 that no subgraph isomorphism *ϕ *between *q *and *g *can map *v *into *v'*. This means that the pair (*v*, *v'*) cannot be involved in any match.

SING solves both the First_query_occurrence and the All_query_occurrences problems. In the First_query_occurrence case, the matching algorithm stops when the first match of the graph is found.

## Authors' contributions

RD, AF, RG, MM, AP, and DS designed, analyzed, implemented and tested the proposed algorithm and contributed in writing the paper. All authors read and approved the final manuscript.

## Supplementary Material

Additional file 1 **sing tool.tar**. sing_tool.tar contains the binaries of SING for Windows and Linux and a sample dataset. Additional files are available at http://ferrolab.dmi.unict.it/. Click here for file
